# Clinico-Radiological Evaluation of Modified Stoppa Approach in Treatment of Acetabulum Fractures

**DOI:** 10.7759/cureus.10193

**Published:** 2020-09-02

**Authors:** Surya V Singh, Rajesh K Chopra, Gyanendra Puri, Mozammil Pheroz, Sapan Kumar, Amit Bansal, Siddharth Gupta, Simarjot Sodhi, Pritam Samanta

**Affiliations:** 1 Orthopaedics and Trauma, Central Institute of Orthopaedics, Vardhman Mahavir Medical College (VMMC) & Safdarjung Hospital, Delhi, IND

**Keywords:** modified stoppa approach, ilioinguinal approach, acetabulum fractures, pelvic trauma, kocher-langenbeck approach

## Abstract

Background

The aim of our study was to evaluate the efficacy of Modified Stoppa approach for surgical treatment of acetabulum fractures by analyzing clinical and radiological outcomes. Also, we assess intraoperative and postoperative complications of fracture treated by using Modified Stoppa approach.

Objectives

To evaluate clinical outcomes of Modified Stoppa approach by using Merle d'Aubigne hip score. To evaluate the radiological reduction quality of Modified Stoppa approach by using the criteria of Matta, and to assess complications of Modified Stoppa approach.

Method

Thirty-two patients participated in this study (mean age 40 years; range 18-60 years) and the male to female ratio was 4:1, patients who underwent surgical intervention for acetabular fracture by using Modified Stoppa approach from Oct 2017 to April 2019 were included. Out of 32 patients, two were lost in follow up, leaving 30 of 32 patients for clinico-radiological analysis. We classified the fracture pattern according to Judet and Letournel classification based on preoperative X-ray AP view, Judet View, and 3D-CT pelvis. Operative time, blood loss, reduction quality, and perioperative complications were assessed in each patient. Clinical outcomes were assessed by Merle d’Aubigne hip score and radiological outcomes by criteria of Matta.

Results

Out of 30 acetabulum fractures in 30 patients, three (10%) were categorized as anterior column fracture, one (3.3%) as transverse with posterior wall, one (3.33%) as T-type, six (20%) as anterior column with posterior hemi-transverse and 19 (63.33%) as both column fractures. In our study, most patients have trauma due to road traffic accident (RTA) in 25 (83.3%) and fall from stairs in three (10%) patients. Timing of surgery after trauma was average 5.83 days (range three to 15 days), Mean surgical time determined to be 214.66 min (range 150-350 min) and mean intraoperative loss 683.33 ml (range 230-1250 ml). Clinical outcomes by Merle d’Aubigne hip score was excellent in 13 (43.33%), good in 15 (50%), fair in two (6.66%) patients whereas poor results in 0 (0%) patient (p=0.001). Quality of reduction by Matta criteria was found to be an anatomical reduction in 26 (86.6%), imperfect reduction in three (10%), and poor reduction in one patient (3.33%) (p<0.001). Radiological grading by Matta criteria was excellent in 24 (80%), good in five (16.66%), and fair in one (3.33%) patient, and no patients met criteria for poor results (p<0.001). In operative complications one patient developed an external iliac vein injury which was repaired by a vascular surgeon, one patient had a superficial infection for which debridement, regular dressing, and IV antibiotics given and resolve in one month, obturator nerve injury in one patient which was resolve in five to six months, lateral femoral cutaneous nerve injury in one patient which resolved within three months and one patient urinary bladder injury which was repaired by a general surgeon.

Conclusion

Our experience with Modified Stoppa approach for surgical treatment of acetabulum fracture in 30 patients is excellent and effective for better visualization to anterior column, quadrilateral plate, and up to sacroiliac joint. This approach provides better visibility of the fracture site which allows for good to an excellent reduction of fracture and fixation. Although Stoppa approach is cosmetic surgery in terms of scar size, there is less complication rate than the ilioinguinal approach.

## Introduction

The acetabulum is the most important weight-bearing joint, the hip joint, and the fracture of the acetabulum is an intra-articular fracture, and to obtain the most favorable results, accurate anatomic reduction, firm fixation, and early rehabilitation are mandatory [1-3].​​ However, the treatment of acetabular fractures is quite difficult not only due to the associated major organ injuries but also due to the complicated fracture type and difficulties in the operative approach for reduction.

Since the initial work of Letournel, the operative treatment of acetabular fractures has become the gold standard for displaced and unstable fracture patterns [4]. Surgical decision making entails fracture classification and the surgical approach. The choice of surgical approach depends on the fracture pattern, direction of displacement, skin condition at the surgical incision site, and duration since the time of injury. Rigid internal fixation is obtained by using several combinations of plates and screws after careful preoperative planning. The pattern of fractures involving medial displacement especially the acetabular quadrilateral plate is technically strenuous, because of the location of the fracture in the true pelvis, the scanty bone stock, and the fracture’s proximity to the articular surface of the hip joint [5].

Several approaches are well explained in the literature and include the Kocher-Langenbeck (KL), Iliofemoral, Ilioinguinal, Combined Anterior and Posterior approaches, Extended Iliofemoral, Transtrochanteric, and Triradiate approaches [6]. These approaches are generally classified as one of limited or extensile, based on the degree of exposure experienced; or anterior or posterior/intrapelvic or extrapelvic depends on the specific zone of the acetabulum exposed. The use of an intrapelvic approach persuades sufficient exposure of the pelvic ring, thereby facilitating the reduction of the anterior wall and column fractures, anterior fractures along with a posterior hemitransverse component, as well as both column fractures. Besides, for some fracture patterns, and intrapelvic approach allows for the utilization of plating configurations that are not possible with an extrapelvic approach [3].

The ilioinguinal, first described by Letournel in 1961, is the only intrapelvic approach of the above-introduced approaches [7]. The use of this approach exposes the anterior column and part of the wall, allowing a limited view of the quadrilateral plate. This approach is indicated in all anterior column and wall fractures, associated anterior column and posterior hemitransverse fractures, selected both column fractures, transverse, and T fractures. It is also recommended for the restoration of displaced superior rami fractures. Its main limitations include a lack of direct visualization of the acetabular surface and a lack of control in extensively displaced posterior column fractures. The main complications of the ilioinguinal approach are a high rate of postoperative infections and iatrogenic injury to the iliofemoral blood vessels and the femoral nerve [8,9].

Along with the evolution of minimally invasive techniques aimed at minimizing surgical dissection, attempts have been made to treat acetabular fractures with an even less extensile approach i.e., the Modified Stoppa (MS) approach. The important difference between the ilioinguinal approach and the Modified Stoppa approach was the avoidance of the ‘‘middle window’’ so that avoiding the dissection of the inguinal canal, femoral nerve, and external iliac vessels. This modified approach provides clear acetabular access including access to the pubic body, pubic tubercle, superior ramus, the ilium above and below the pectineal line, the quadrilateral plate, the medial aspect of the posterior column, sciatic buttress, and the anterior sacroiliac joint.

Stoppa described his approach first in 1973 as a subperitoneal median approach for the treatment of groin hernias with the support of a Dacron tulle prosthesis [10]. In 1994, Cole and Bolhofner proposed the Stoppa approach in which the surgeon to stand at the opposite side of the involved hip joint during reduction and fixation of the acetabular fractures, allowing for direct visualization of the medial wall, dome, quadrilateral plate, and extending as far as the sacroiliac joint [11]. Nevertheless, this approach does not enable maneuvering the fractures for anatomical reduction of the iliac wing, a necessary step in the anatomic restoration of the anterior wall. 

Therefore, along with the Stoppa approach, an additional lateral iliac wing window is necessary to achieve this surgical task. Jakob et al. and recently, Anderson et al. Illustrate a modified approach using the Stoppa and iliac surgical window for the treatment of acetabular and pelvic ring fractures [12,13]. This approach became popular over the past decade for anterior column fractures and is satisfactory for the majority of cases while providing excellent visualization of and good entrance to the quadrilateral plate and parts of the posterior column [11]. 

We present this Modified Stoppa approach with a lateral iliac window, enroll the suprapubic and lateral windows that are partially utilized in the ilioinguinal approach. This allows the excellent visualization of the entire anterior column, quadrilateral plate, and the pelvic brim portion of the posterior column, previously concealed in the ilioinguinal window [14-16].

## Materials and methods

After obtaining a detailed history, complete clinical examination, the patients were subjected to relevant investigations like pre-operative X-ray, 3D CT scan of the pelvis to compare similar fracture patterns. Finally, after the diagnosis, the patients were selected for study depending on inclusion and exclusion criteria. 

The duration of the study was 18 months, from October 2017 to April 2019. Our study design was a Prospective/Therapeutic level 2 study. The total sample size was 32 cases in which two cases were lost in follow up. Study population: all patients of age between 18-60 years with complaints of traumatic injury leading to fracture acetabulum, visiting in the emergency room (ER), Central Institute of Orthopaedics, Safdarjung Hospital were recruited to participate in the study (Table 1).

**Table 1:** Demographic characteristics of the patients. RTA: road trafiic accident

Variables		Values ( percent)
Age group		Mean age 40 years, range 18 to 60 years
Sexes (M: F)		4:1
Mode of Injury	Fall from height	2 (6.7%)
	Fall from stairs	3 (10%)
	RTA	25 (83.3%)
Time of surgery after trauma		5.83 days (3-15 days)
Fracture types	Anterior column	3 (10%)
	Transverse + posterior wall/ column	1 (3.33%)
	T-type	1 (3.33%)
	Anterior column + posterior hemitransverse	6 (20%)
	Both column	19 (63.33%)
Operative Time (min)		214.66 ( range 150 – 350 min )
Blood loss (ml)		683.33 ( range 230 - 1250 ml )
Blood transfusion (in units)		1.8 ( 1 – 3 units )

Criteria for inclusion

1) Age 18 to 60 years, 2) all sexes, 3) Acetabulum fracture with involvement of anterior column, quadrilateral plate, t-type, bicolumnar, and anterior column or wall fractures associated with a posterior hemitransverse component. 

Criteria for exclusion

1) Sciatic buttress comminuted fracture, 2) Fractures greater than three weeks old, 3) Patients with a suprapubic catheter, 4) Ipsilateral lower limb fracture of affected hip, 5) General comorbidity like Head injury, poor general medical condition. 

Preoperative assessment

**Figure 1:**
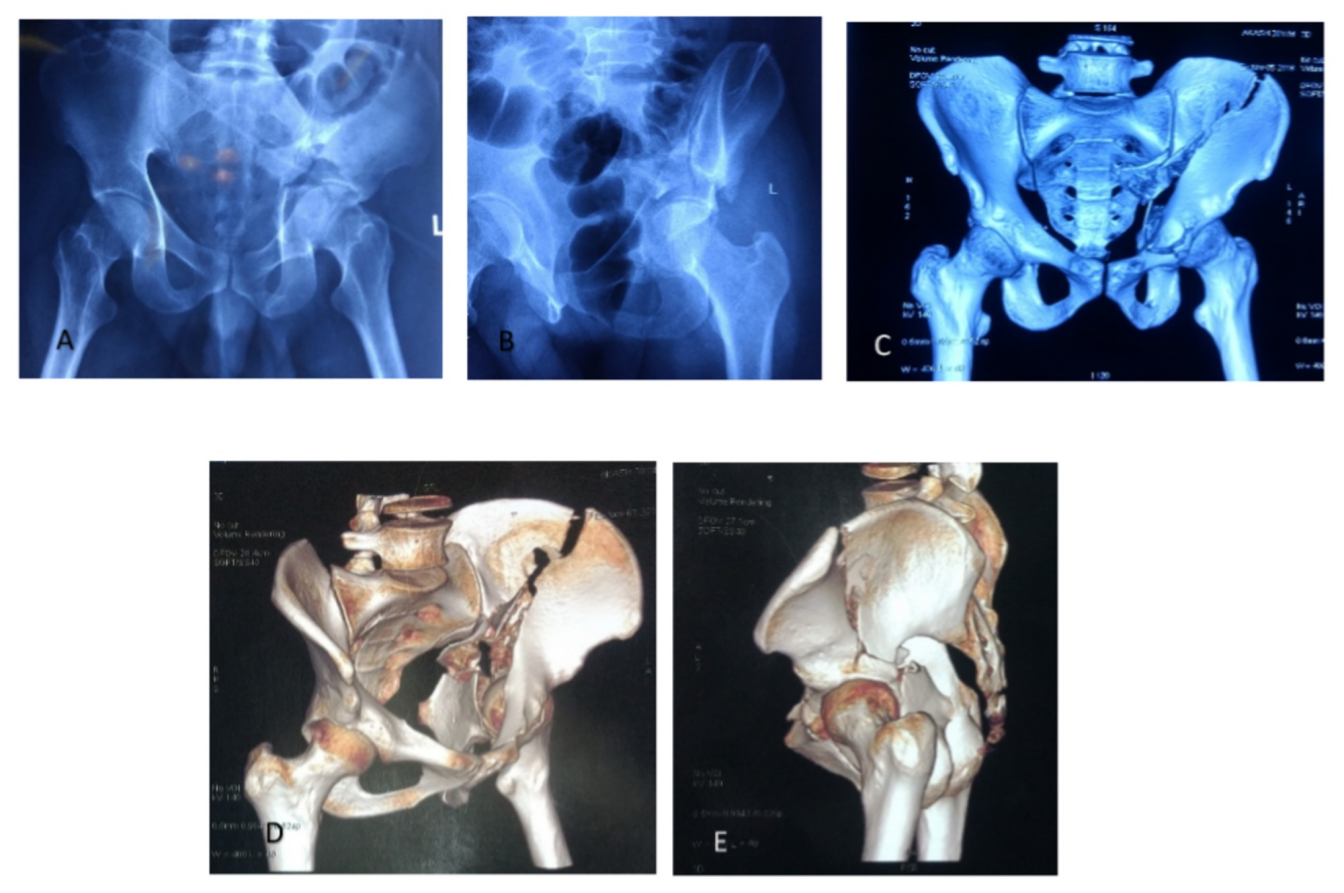
Preoperative x-rays and 3D-CT pelvis showing both-column fracture of left acetabulum: A) Anterior posterior view radiograph of pelvis; B) obturator view radiograph of pelvis; C,D,E) 3D-CT pelvis.

After obtaining a detailed history, complete clinical examination, the patients are subjected to relevant investigations. The patient is subjected to pre-operative X-ray hip with the pelvis (AP and judet view), 3D CT scan of the pelvis in an effort to compare similar fracture patterns. All fracture is classified according to Judet and Letournel classification (Figure 1).

Preoperative skeletal traction, antibiotics, and part preparation are done. Urinary catheterization is done before surgery. The patient is positioned supine on the operating table, Approximately 20° elevation of the contralateral side and the reducibility of the fracture is checked with C-arm. Surgeons stand on the opposite side of the fracture to allow better visualization and good access to the true intrapelvic cavity.

Surgical technique 

**Figure 2:**
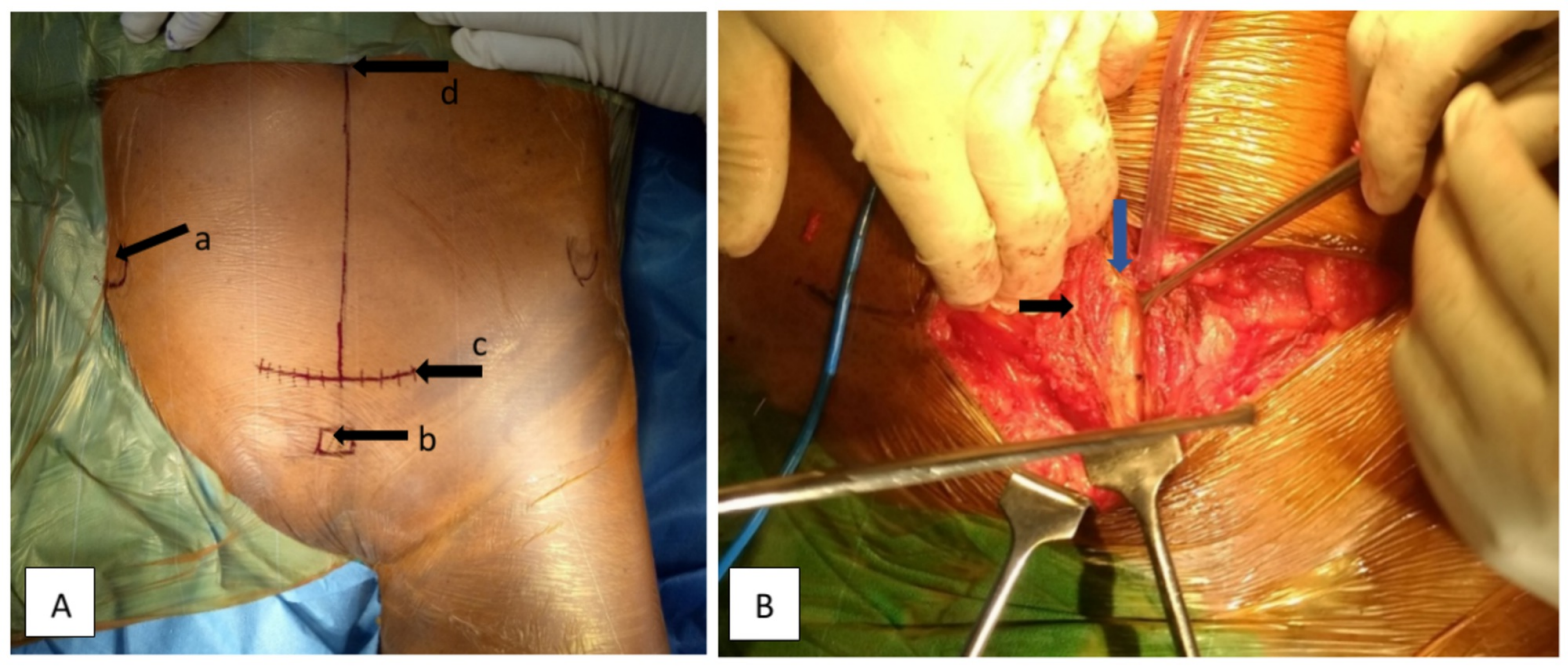
A) Patient is lying supine on OT table showing [with black arrow] a) anterior superior iliac spine; b) pubic symphysis; c) site of transverse skin incision 2 cm proximal to the symphysis pubic; d) umbilical. B) intraoperative intrapelvic modified stoppa approach shows layers of abdominal wall; linea alba (blue arrow), rectus abdominis muscles (black arrow).

The surgical skin incision is made 1-2 cm proximal to the symphysis pubis in the transverse fashion of size 8-10 cm also known as Pfannenstiel incision (Figure 2A). Our dissection is made through the skin and subcutaneous fascia. The rectus abdominis muscles are dissected and split vertically from symphysis pubis inferiorly to continued superiorly 3-4 cm distal to the umbilicus, and transversalis fascia is incised just superior to the pubic symphysis (Figure 2B).

Sharp subperiosteal dissection of the rectus abdominis is carried out along the symphysis pubis, pubic tubercle, and superior pubic ramus. Blunt dissection of the space of retzius is performed and exposing the symphysis body and pubic ramus. Deep dissection starts at the side of the fracture of the symphysis pubis and gradually “crawls” posteriorly on the pelvic brim, towards the ipsilateral sacroiliac joint. A pointed Hohmann retractor is placed over the pubic tubercle to retract the rectus muscle.

**Figure 3:**
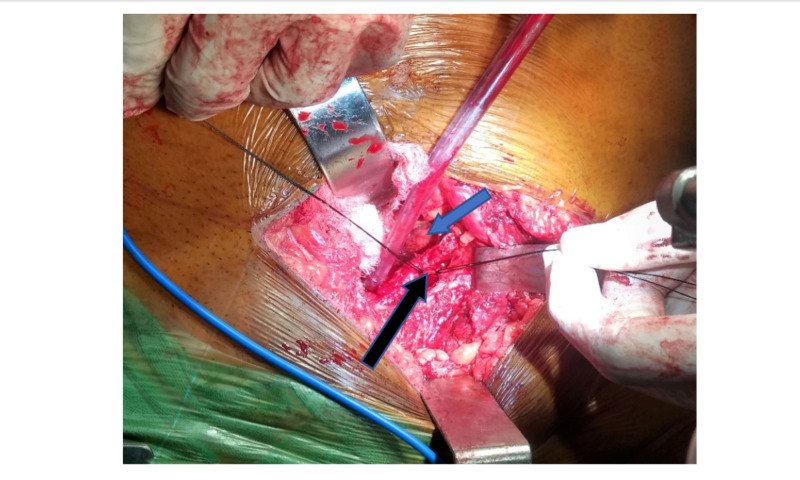
Intraoperative intrapelvic Modified Stoppa Approach showing Corona Mortis (black arrow) and urinary bladder (blue arrow).

Along the first one-third course of the pelvic brim, in the majority of the cases, the Corona Mortis artery is present, which is an anastomotic branch between the internal iliac branch (obturator artery) and external iliac artery or its branch, the inferior epigastric artery. It is also known as "Crown of Death". It is found in a variable location between 3-7 cm from the pubic tubercle over the pelvic brim. The incidence of Corona Mortis is about 84% in cadaveric studies. It is more common in females and the venous connection is more prevalent. We ligated the corona Mortis artery and vein separately using silk sutures or vascular clips after dissection and identification of the Corona Mortis vessel (Figure 3). 

At the second third of the pelvic brim, a sharp incision of the iliopectineal fascia from the obturator fascia, this incision is carried out along the interrupted line, and then elevation of both fascia is carried out and the medial and anterior wall of the acetabulum is exposed. With blunt dissection identify the obturator nerve which arises from the lateral part of a large part of the sacrum and enters the obturator foramen and it should be preserved. The blunt Hohmann retractor is then placed at the lesser sciatic notch with retracting urinary bladder and a second sharp retractor should be advanced and placed at the anterior column and fixed by hammering its tip to the bone, just proximal to the acetabular roof. Once the latter retractor is used, special attention should be paid to the femoral vein and nerve, while elevating the abdominal muscles, as they lay in close proximity to the retractor. The blunt retractor is advanced from the lesser sciatic notch to the greater, so that at this stage of dissection, full access is present anterior to posterior along the pelvic brim, sharply dividing and elevating the iliopectineal fascia superiorly and the obturator fascia inferiorly, exposing the medial wall of the acetabulum.

Complete access to the sacroiliac joint can be obtained by dissecting posteriorly, for this; the use of a sharp retractor placed at the sacroiliac joint is useful. Elevation of the psoas muscle further allows access to the sciatic buttress and posterior aspect of the pelvic brim. The quadrilateral plate and part of both the anterior and posterior columns will be under direct observation. This view is not to be obtained with the ilioinguinal approach. At this stage, a Hohmann retractor is placed in the greater sciatic notch, with special caution paid to the neurovascular structures. At this juncture, the surgeon should be able to visualize the notch, the ischial spine, and the quadrilateral plate.

**Figure 4:**
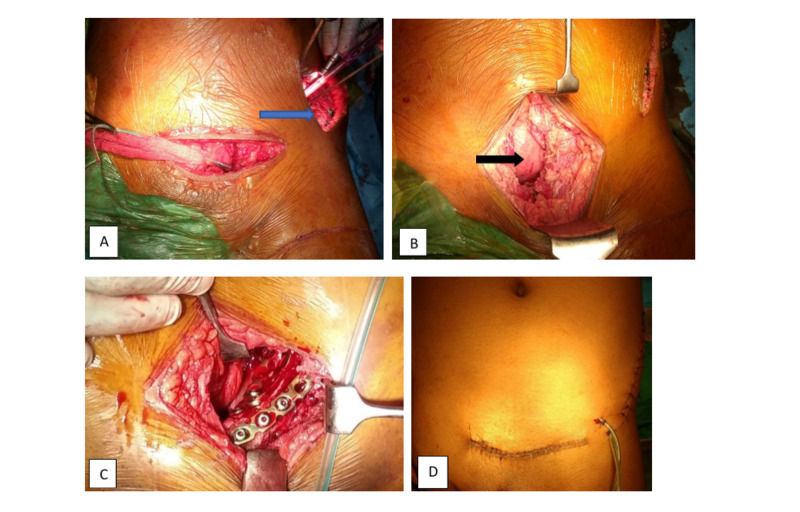
A) Intraoperative Modified Stoppa approach showing the lateral window for iliac wing fracture ( blue arrow ), B) urinary bladder ( black arrow ). C) After reduction of anterior column and iliac blade plate fixation done and D) postoperative drain placement is done in the space of Retzius and lateral window if used.

A reconstruction plate is placed across the transverse fracture together with posterior column screws under observation. The obturator nerve and vessels are retracted medially with a malleable retractor. Reconstruction plates are placed across the fracture line in different configurations. This is only possible when using the Stoppa approach, these plates are a medial buttress of the quadrilateral plate. Access to the iliac wing for fractures is available through the lateral window of the ilioinguinal approach. This window involves an incision along the superior border of the iliac wing with release and reflection of the abdominal muscles proximally; submuscular dissection along the internal iliac wing allows access to most anterior column fractures and provides additional access for plate and screw placement. Postoperative, drain placed in the space of Retzius (and lateral window, if used). Suturing done in layers, rectus muscle sutured tightly with absorbable vicryl and skin with nylon suture or staples (Figure 4).

Postoperative management

**Figure 5:**
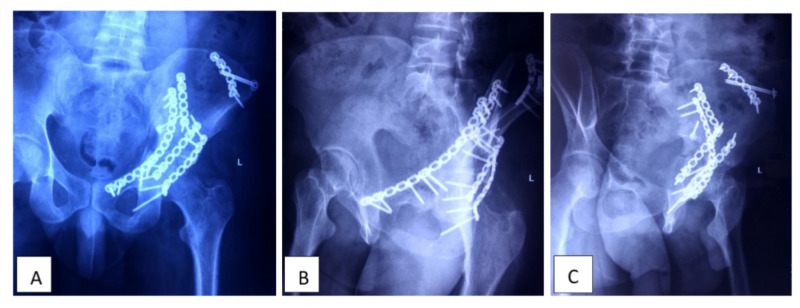
Postoperative radiographs of the patient’s hip with pelvis; A) anterior posterior view, B) obturator view, C) iliac oblique view of hip with pelvis. *additional kocker-langenbech posterior approach required if posterior acetabular wall /column fracture.

The patient is subjected to a postoperative x-ray (AP and judet view). Postoperative IV antibiotics and analgesic is started. Drain removal is performed when less than 30 ml of drainage over a 24 hours period. Because postoperative ileus is common, careful postoperative monitoring of the patient, and slow advancement of diet. Bowel sounds and the presence of flatus guided the advancement of the diet. Depending on the associated injury and patient stability, mobilization with physical therapy on the bed is starting on post-op day one, and suture removal will be done after 15 days (Figure 5). 

Physical therapy on 1st postoperative day with early out of bed mobilization and the involved extremity hip abductor and extension exercises are encouraged throughout follow up. Weight-bearing restrictions for six weeks postoperatively followed by partial weight bearing until full weight-bearing at 12 weeks. Postoperative clinical and radiological assessment was done after month one, three, and six by Criteria of Matta and Merle d'Aubigne hip score.

Statistical testing was conducted with Statistical Product and Service Solutions (SPSS), version 21 (IBM Corp., Armonk, NY). Continuous variables are presented as mean ± SD, and categorical variables are presented as absolute numbers and percentages. The comparison of normally distributed continuous variables between the groups was performed using Student’s t-test. P<0.05 was considered statistically significant.

## Results

A total of 32 patients were considered for this study and after implementation of the exclusion and inclusion criteria, two patients were lost in follow up, so a total of 30 patients were considered for this study. Out of the total 30 patients in this study, 11 (36.7%) were in the age group of 31 to 40 years; 10 (33.3%) patients were in the age group of 41 to 50 years; six (20.0%) patients were in the age group of 21 to 30 years; whereas only three (10.0%) patients were in the age group of 51 to 60 years. The mean age of the study group was 40 years with a range of 18 to 60 years. The majority of the patients (24, 80.0%) were males whereas only six (20.0%) were females. The distribution of the study group showed a male preponderance with male to female ratio of 4:1. In our study, all the patients suffered high energy trauma, out of the 30 patients, the majority (25, 83%) patients had an injury due to Road Traffic Accidents (RTA); three (10.0%) patients had an injury due to fall from stairs, and the last two (7.0%) were due to fall from height (Table 1).

Based on Judet and Letournel classification system, the 30 acetabulum fractures of the 30 patients were categorized three (10%) as anterior column, one (3.3%) as transverse with posterior wall, one (3.3%) as T-type, six (20%) as anterior column with posterior hemitransverse and 19 patients (63.33%) as both column fracture. Classification of the acetabulum fractures according to Judet and Letournel system was done by an operating surgeon and was also confirmed preoperatively by a radiologist experienced in extremity imaging. In our study, out of 30 patients, 15 (50%) patients have operated with Modified Stoppa (MS) approach, and rest 15 (50%) patients required fixation from posteriorly (Kocher-Langenbach (KL) approach) intraoperatively so in 15 patients along with Modified Stoppa approach additional firm fixation done with KL approach. In the study population, the timing of the operation was an average of 5.83 days (range 3-15 days). All the operations were performed by a single surgeon. The mean surgical time determined to be 214.66 min (range 150-350 min). The mean intraoperative blood loss was determined to be 683.33 ml (range 230-1250 ml). The mean intraoperative blood transfusion is determined to be 1.8 units (range 1-3 unit).

Out of 30 acetabulum patients, function hip evaluation done by Merle d’Aubigne hip score was excellent in 13 (43.33%) patients, good in 15 (50%) patients, and fair in two (6.66%) patients, whereas no poor results were seen. After six months of follow up, clinical outcomes were statistically significant (p<0.001 ). Radiological grading by Matta criteria was excellent in 24 (80%) patients, good in five (16.66%), and fair in one (0.33%), whereas no patients showed poor results. The correlation between radiological outcomes and the operative procedure was statistically significant (p<0.001). Quality of reduction on radiological grading by matta criteria, the anatomical reduction in 26 (86.6%) patients, imperfect reduction in three (10%) patients; and poor reduction in one patient (3.33%) due to infection. The relationship between quality of reduction and clinical outcomes is statistically significant (p<0.001) (Table 2).

**Table 2:** Clinico-radiological variables with results.

Variables	Results
Clinical grading by Merle d’aubigne hip score	
Excellent	13 ( 43.33%)
Good	15 ( 50%)
Fair	2 (6.66%)
Poor	0 (0%)
Radiological grading by matta criteria	
Excellent	24 ( 5%)
Good	5 (16.66%)
Fair	1 (3.33%)
Poor	0 (0%)
Reduction quality by matta criteria	
Anatomical reduction ( < / =1)	26 ( 86.66 %)
Imperfect reduction ( >1 to <3 mm)	3 (10 %)
Poor reduction ( >/= 3 mm)	1 (3.33%)

Intraoperatively, one patient has developed an external iliac vein injury which was repaired by the cardiovascular surgeon and one patient has developed a urinary bladder injury which was repaired by a general surgeon, postoperatively one patient developed a superficial infection, one patient has obturator nerve palsy, and one patient has lateral femoral cutaneous nerve injury. For superficial wound infection was successfully treated with debridement and prolonged antibiotic therapy. Obturator nerve palsy and lateral femoral cutaneous nerve palsy were spontaneously resolved in three to five months. No postoperative ileus has developed following surgery. No rectus abdominis muscle paralysis was noted postoperatively. No incisional or groin hernias were developed after surgery (Table 3).

**Table 3:** Operative complications of Modified Stoppa Approach.

Variables	Results
Operative Complications	
None	25 (83.3 %)
External iliac vein rent	1 (3.33 %)
Superficial infection	1 (3.33 %)
Obturator nerve palsy	1 (3.33 %)
Lateral femoral cutaneous nerve of thigh palsy	1 (3.33 %)
Urinary bladder rent	1 (3.33 %)

## Discussion

Age and gender distribution

In our study, most of the patients included in the study were males (24, 80%) and the male to female ratio was 4:1. Most of the 11 (36.7%) patients were in the middle age group between 31 to 40 years and secondly 10 patients (33.3%) were in the age group 41 to 50 years. The mean age of the study group was 40 years with a range of 18 to 60 years. The majority of the patients 24 (80.0%) were males whereas only six (20.0%) were females. The distribution of the study group showed a male preponderance with a male to female ratio of 4:1.

A similar study by Liu et al. in 2013 conducted on 29 patients showed the mean age group of 42.3 years and the male-female sex ratio was 22:7 [17]. A similar study by Isaacson et al. in 2014, conducted on 36 patients, showed a mean age group of 46.5+/-16.1 years with a range from 18 to 87 years. Most of the patients were male (31, 86.1%) [18]. A similar study by Kim et al. from 2007 to 2010 on 22 patients showed an average age of 20-74 years with a male/female ratio of 8:3 [19]. Also the study by Verbeek et al. in March 2018, conducted on 45 patients, showed a mean age group of 51 years and a male preponderance of 71%. The majority of the sex distribution was male 36 (63.157%) and the male-female ratio was 36:21. Hence, it is clear that the trauma to acetabulum is more common in young and middle age group males, who are the most productive age group of the society, producing a major economic burden on society. Therefore it is necessary to provide them with the best possible method of treatment with less postoperative complications.

Distribution of etiology

In our study, most of the patients had an acetabular fracture due to RTA (25, 83.3%) or fall from stairs (three, 10%), which are the two most common causes for acetabulum fracture.

Also in the study by Liu et al. in 2013 conducted on 29 patients shows that RTA 17 patients (58.6%) then fall from height nine patients (31.0%) were mostly the mode of injury [17]. A similar study by Verbeek et al. in 2018 conducted on 45 patients shows that high energy trauma due to 69% RTA causes an acetabular fracture. Almost most of the studies involving the acetabular fracture have reported road traffic accidents and fall from height as a common cause of the acetabular fracture.

Approach

In our study, out of 30 patients, 15 (50%) patients were operated with the Modified Stoppa approach, and the rest 15 (50%) patients required fixation from posteriorly KL approach intraoperatively so in 15 patients along with Modified Stoppa approach addition firm fixation given with KL approach.

A similar study by Isaacson et al. in 2014 on 36 patients shows that the use of a supplementary posterior KL in three patients (8.3%) for an additional posterior column or wall fracture [18]. Also in a study by Cole and Bolhofner in 1994 on 55 acetabular fracture in which he used only Modified Stoppa anterior intrapelvic approach in 32 (58%) patients and utilization of posterior approach (KL) in 18 (33%) patients [11]. Almost the majority of our studies Modified Stoppa approach is taken for the all fracture pattern in inclusion criteria and KL approach required only in those cases where rigid fixation needed from posteriorly along with Stoppa approach. So the Modified Stoppa approach can be combined with the KL approach, lateral window, and medial window of the ilioinguinal approach in various studies.

Distribution on the basis of classification of the acetabulum

In our study, on the basis of Judet and Letournel classification system, the 30 acetabulum fractures of the 30 patients were categorized as anterior column in three patients (10%), transverse with posterior wall in one patient (3.3%), T-type in one patient (3.3%), anterior column with posterior hemitransverse in six patients (20%) and both column in 19 patients (63.33%).

Also in a study by Liu et al. in 2013 conducted on 29 patients shows that the acetabular fracture classified on the basis of Judet and Letournel classification system, which includes anterior column in 10 patients (34.5%), transverse type in four (13.8%), T-type in six (20.7%), anterior column with posterior hemitransverse in seven (24.1%) and both column in two (6.9%) [17]. Also in a study by Isaacson et al., in January 2014 conducted on 36 patients shows that the anterior column (2.8%), transverse with posterior wall (5.6%), T-type (16.7%), anterior column with posterior hemitransverse (19.4%), both column (41.7%) and transverse type (13.9%) [18]. In a study by Verbeek et al., in March 2018 conducted on 45 patients shows that the anterior wall (3%), transverse (8%), T-type (16%), anterior column with posterior hemitransverse (5%), and both column (68%). Almost the majority of studies show that both column fractures are the most common fracture type. In our study also both column fractures are most common (63.33%).

Intraoperative blood loss, surgical duration, and blood transfusion

In our study population, the timing of the operation was an average of 5.83 days (range 3-15 days). All the operations were performed by a single surgeon. The mean surgical time determined to be 214.66 min (range 150-350 min) whereas the mean surgical time for only Modified Stoppa was 185.33 min and for MS+KL was 244.00 min. The mean intraoperative blood loss was determined to be 683.33 ml (range 230-1250 ml) whereas mean intraoperative blood loss by only Modified Stoppa was 494.00 ml and MS+KL was 872.67 ml. The mean intraoperative blood transfusion determined to be 1.8 units (range 1-3 units).

A similar study by Sagi et al. in 2010 conducted a study on 57 patients on anterior intrapelvic (Modified Rives-Stoppa) approach for fixation of acetabular fractures shows that the average intraoperative blood loss was 750 ml and the average surgical time in minutes was 263 [15]. Also in a study by Fan et al. in 2012 conducted on 16 patients with Modified Stoppa approach in the treatment of pelvic and acetabular fractures shows that the average surgical time was 130 minutes (range 50 to 350 minutes) and the average intraoperative blood loss was 320 ml (range 100 to 1200 ml) [16]. A similar study by Wanget al. in 2010-2015 reviewed eight studies on 637 patients shows that median intraoperative blood loss was 199 ml, and the average operative time 302 min [20]. A study by Kim et al. in 2017 conducted on 22 patients shows that obturator nerve injury intraoperatively 2/22 (9.1%) by Modified Stoppa approach [21]. Also in a study by Liu et al. in May 2013 conducted on 29 patients on newly Modified Stoppa approach for acetabular fractures in which the mean surgical time 155 minutes (range 90 to 270 minutes), average intra-operative blood loss 950 ml (range 600 to 1800 ml) and average blood transfusion was two units (range 1-5 units) [17]. Also in a similar study by Isaacson et al. in January 2014 conducted on 36 patients shows that the Treatment of Acetabulum Fracture through the Modified Stoppa Approach in which the average intraoperative blood loss in ml was 1041.4 +/- 946.7 (range 100 to 5000), mean surgical time (minutes) was 320.2 +/- 81.8 (range 199 to 568 minutes) and average blood transfusion in units was 1.9 +/- 1.8 (range 0 to 6) [18]. Also in a study by Zhang et al. in 2017 conducted on 36 patients on minimally invasive treatment of both-column acetabular fractures through the Stoppa combined with iliac fossa approach shows that the intraoperative blood loss in ml was 466.67 +/- 169.33 and mean surgical time in minutes was 84.17 +/- 16.32 [22]. A similar study by Verbeek et al. in March 2018 conducted on 45 patients in a retrospective study on the Modified Stoppa Approach for operative treatment of acetabular shows that the average intraoperative blood loss in ml was 1850, mean surgical time (minutes) was 221 and average blood transfusion in units was two [23].

Clinical outcomes by Merle d’Aubigne hip score

In our study, out of 30 acetabulum patients, Merle d’Aubigne hip score was excellent in 13 (43.33%), good in 15 (50%), and fair in two (6.66%) whereas no poor results were seen. After six months of follow up, clinical outcomes were statistically significant (p=0.001).

A similar study by Sagi et al., in 2010 on 57 patients' clinical outcomes (Merle d’Aubigne hip score) was excellent in 36%, good in 55%, and poor in 10% cases [15]. A similar study by Isaacson et al. in 2014 on 36 patients on Treatment of Acetabulum Fracture through the Modified Stoppa approach results shows that clinical outcomes as per Merle d’Aubigne hip score were excellent/good in 82% cases, fair in 5% cases and poor in 14% cases [18]. Similarly in a study by Verbeeket et al. in 2018 on 45 patients shows results that Merle d’Aubigne clinical outcomes were excellent in six (35%) patients, good in nine (53%) patients, and fair results in two (12%) [23]. A study by Matta et al., on 204 acetabular fractures concluded that fracture must be reduced up to <=3 mm to a congruent reduction of the hip joint at the weight-bearing dome of acetabulum [24]. Also in a study by Cole and Bolhofner in 55 acetabular fracture in 1994 where clinical scoring excellent in 47%, good in 42%, fair in 9%, and poor results in 2% acetabular fracture [11]. In the majority of the study, the clinical outcomes according to Merle d’Aubigne hip score are excellent and good and a correlation between clinical outcome and Modified Stoppa approach is statistically significant (p=0.001).

Quality of reduction by Matta criteria

Out of the 30 patients, quality of reduction on radiological grading by Matta criteria, the anatomical reduction in 26 (86.6%) patients, imperfect reduction in three (10%) patients; and poor reduction in one patient (3.33%) due to infection. The relationship between quality of reduction and clinical outcomes is statistically significant (p<0.001).

Also in a study by Liu et al. in 2013 conducted on 29 patients on a newly Modified Stoppa approach for acetabular fractures in which the quality of reduction was anatomical in 24 (82.8%), imperfect in four (13.8%), and poor in one (3.4%) [17]. A similar study by Isaacson et al., in 2014 on 36 patients in which the quality of reduction was anatomical/satisfactory in 92% cases by Modified Stoppa approach [18]. Also in the study, Kilinc et al. in 2018 on 57 patients where the quality of reduction was anatomical (<=1) in 84%, imperfect (>1 to <3) in 12.7% cases, and poor (>=3) in 3.2%. Similarly in a study by Verbeek et al. in 2018 on 47 acetabular shows results that the quality of reduction was an anatomical reduction in 23, imperfect in 16, and poor results in eight acetabulum fractures [23]. In the majority of the study, the quality of reduction is anatomical, and the correlation between reduction quality and clinical outcomes is statistically significant (p<0.001).

Radiological grading by Matta criteria

Out of the 30 acetabulum patients, radiological grading by Matta criteria was excellent in 24 (80%) patients, good in five (16.66%), fair in one (0.33%) whereas no patients showed poor results. The correlation between radiological outcomes and the operative procedure was statistically significant (p<0.001). A similar study by Isaacson et al., in 2014 on 36 patients in which the radiographic grading was excellent in 27 (75%) cases, good in six (17%), and poor in 8% cases [18]. A similar study by Sagi et al. in 2010 on 57 patients in which radiological outcomes were excellent in 70%, good in 22%, and poor in 10% [15]. Also in a study by Cole and Bolhofner in 55 acetabular fracture in 1994 by a Modified Stoppa approach for acetabular fracture where radiological grading shows that good in 84%, fair in 9%, and poor in 7% [4,11]. Hence, it is clear that most of the study shows excellent and good radiological outcomes by the Modified Stoppa approach; and the correlation between that is statistically significant (p<0.001).

Operative complications of Modified Stoppa approach

In our study, out of the 30 patients, one patient has developed an external iliac vein injury which was repair by a vascular surgeon, one patient (3.33%) has the superficial infection for which debridement, regular dressing, and intravenous antibiotics were given, and resolve in one month, one patient (3.33%) developed obturator nerve injury and resolved in five to six months, one patient (3.33%) developed lateral femoral cutaneous nerve injury which was resolved within three months, and one patient (3.33%) developed urinary bladder injury intraoperatively which was 3.33% that was repaired by a general surgeon.

Isaacson et al. in 2014 conducted a study on 36 patients on Treatment of Acetabulum Fracture through the Modified Stoppa Approach where developed intraoperative complication like lateral femoral cutaneous of the thigh in two patients (5.6%) but no medial thigh numbness and developed a postoperative superficial infection in a single case (2.7%) and deep infection in three patients (8.3%), also developed deep vein thrombosis (DVT) in three (8.3%) patients [18]. Also in the study by Kilinc et al. in July 2018 conducted on 57 patients by Modified Stoppa approach on acetabular fracture which shows external iliac vein injury in two patients (3.5%), deep infection in two patients (3.5%), obturator nerve injury in three patients and DVT in four patients (7%). Deep infection was reported by Sagi et al. in 1.8%, Anderson et al. in 5.9%, Cole et al. in 1.8%, Hirvensalo et al. in five patients (3%), and Kiline et al. in two patients (3.5%) [11,13-15,25]. It was treated with debridement with irrigation and antibiotic therapy but this results in fixation failure and poor prognostic factors. Obturator nerve palsy was reported by Kiline et al. in three patients which were resumed in 3.7 months, Sagi et al. in 13 patients (26%) which was recovered in six months, Zhang et al. in two patients which recovered in six months [15,22]. A study done by Soni et al., in 2016, reviewed 16 studies total 609 patients were treated and show that the mostly intraoperative complication was obturator nerve palsy (21) which was resolved after three months [26]. A study was done by Elmadag et al. in 2016 on 36 patients shows postoperative foot drop (two patients), obturator nerve injury (one patient), and iliac vein injury (patient) [27]. 

Hence, the majority of the study developed intraoperative and postoperative complications which illustrate above. The intraoperative complication is due to instrumental, surgeons' faults, and patients' comorbidities which can be reduced by using proper autoclaved instruments, surgery is done by a well-experienced surgeon. Postoperative infection can be controlled by using autoclaved instruments, maintaining intraoperative sterility, and postoperative good antibiotics coverage.

## Conclusions

Our experience with the Modified Stoppa approach for the surgical treatment of acetabulum fractures in 30 patients is excellent and effective due to better visualization to anterior column, quadrilateral plate, and up to the sacroiliac joint. As Modified Stoppa approach provides better visualization of fracture sites which help in good to excellent reduction of fracture as well as fixation. In this study for posterior column displacement with anterior column fracture, we used Modified Stoppa with posterior Kocher-Langenbeck approach for good quality of reduction and clinical outcomes. The perioperative complication rate is lesser in Modified Stoppa approach than the widely used ilioinguinal approach. Although the Stoppa approach is extremely cosmetic surgery in terms of scar size, there is less complication rate than the ilioinguinal approach.

We highly recommended the use of a Modified Stoppa approach for fracture acetabulum over the ilioinguinal approach. We also recommend the use of this approach in future studies including a large sample size population, long term follow-up, prospective study, and using a control group would be helpful in further assessing this approach.
